# Staged therapeutic surgery for progressive pulmonary regurgitation and pacemaker induced cardiomyopathy after the tetralogy of fallot repair

**DOI:** 10.1186/s13019-024-02585-2

**Published:** 2024-02-16

**Authors:** Goki Inno, Keiichi Itatani, Kenta Nishiya, Yosuke Takahashi, Toshihiko Shibata

**Affiliations:** 1 Department of Cardiovascular Surgery, Osaka Metropolitan University Graduate School of Medicine, 1-4-3, Asahimachi, Abeno-Ku, Osaka, 545-8585 Japan; 2 Department of Cardiovascular Surgery, Nagoya City University Graduate School of Medical Sciences, Kawasumi 1, Mizuho-cho, Mizuho-ku, Nagoya, 467-8601 Japan

**Keywords:** Cardiac resynchronization therapy, Epicardial lead, Pulmonary valve replacement, Tetralogy of Fallot

## Abstract

**Background:**

Recently, improvements in the repair of tetralogy of Fallot have increased the need for reoperation in adulthood, and it’s not rare that these reoperation candidates suffer from biventricular failure. However, there are no firm treatment guidelines, and each country, and even each facility, treats each case individually.

**Case presentation:**

We report the successful staged treatment of pulmonary regurgitation and pacemaker-induced cardiomyopathy with biventricular failure in adulthood in a case of complete atrioventricular block after tetralogy of Fallot repair in childhood. We planned a staged therapeutic strategy with preoperative left ventricular volume reduction with medication, following surgical pulmonary valve replacement concomitant epicardial lead implantation on the lateral basal wall, placed just beneath the generator pocket through 3rd intercostal space. in addition to postoperative intervention with a defibrillator to adjust cardiac resynchronization therapy, resulted in improvement of symptoms.

**Conclusion:**

In a patient with biventricular failure after TOF repair, a staged treatment strategy involving medication, PVR, and CRT with a combination of epicardial and intravenous leads could be a useful treatment worth trying before heart transplantation.

## Background

Recently, improvements in the repair of tetralogy of Fallot (TOF) have increased the need for reoperation in adulthood, especially for pulmonary regurgitation (PR) [1]. Due to ventricular interaction, right ventricle (RV) dysfunction is associated with a variable degree of LV dysfunction as well [2]. Thus, It’s not uncommon for such these reoperation candidates suffer from biventricular failure. However, the pulmonary valve replacement (PVR) increases left ventricular (LV) preload causing LV dysfunction, which often complicates therapeutic strategies. We reported a patient with PR and pacemaker-induced cardiomyopathy (PiCMP) after TOF repair, who was successfully treated with staged therapy of medication, PVR, and cardiac resynchronization with an additional epicardial lead.

## Case presentation

A 52-year-old male underwent TOF repair at the age of 4 years and permanent pacemaker implantation for complete atrioventricular block (CAVB) at the age of 9 years, resulting in PiCMP caused by long-term dyssynchrony. Furthermore, when he was 48 years old, he underwent cardiac resynchronization therapy (CRT) for dyspnea with a reduced LV ejection fraction (LVEF) of approximately 35%. However, the transvenous lead could not be placed in the optimal position through the coronary sinus due to the access difficulty.

Four years later, progressive PR, which is an independent comorbidity of biventricular failure after the TOF repair mainly caused by PiCM, developed with a heart murmur. One year before the reference to our institution, PR progressed from mild to moderate (Vena Contracta = 3.9 m). At that time, although the right ventricle was small, the fractional right ventricular area change (RVFAC) was 57.5%, so right heart function was preserved. Six months ago, PR progressed further (Vena Contracta = 7.9 m) and his right ventricular function worsened (RVFAC = 48.0%). When he referred to our hospital, PR had progressed to severe range (Vena Contracta Width/pulmonary valve diameter = 0.8) and his right ventricular function had deteriorated further (RVFAC = 42.1%). Furthermore, he suffered from depression one year ago, and antidepressant medication caused QT prolongation with fragmented QRS on an electrocardiogram and weight gain as side effects. we believe that these facts increased cardiac load. Therefore, he was referred to our hospital for surgical treatment. One hypothesis regarding the mechanism of PR progression during these this period It is possible that deterioration of left ventricular function collapsed the pulmonary circulation, putting adding a stress on the pulmonary valve and causing dilatation of the pulmonary valve annulus, even though but there is not sufficient evidences. Echocardiography showed severe PR, and the LV end-diastolic/end-systolic diameters (LVDd/Ds) were 53 mm/38 mm, respectively with LVEF 33%. A catheter hemodynamic study showed an elevated left ventricular end-diastolic pressure (LVEDP) to 17 mmHg.

At the initial stage, we started administration of diuretics and a soluble guanylate cyclase (sCG) stimulator in addition to foundational quadruple therapy for heart failure, to decrease LVEDP. This avoided postoperative lung congestion after the PVR, and 2 months later the LVEDP was finally decreased to 4 mmHg.

**Fig. 1:**
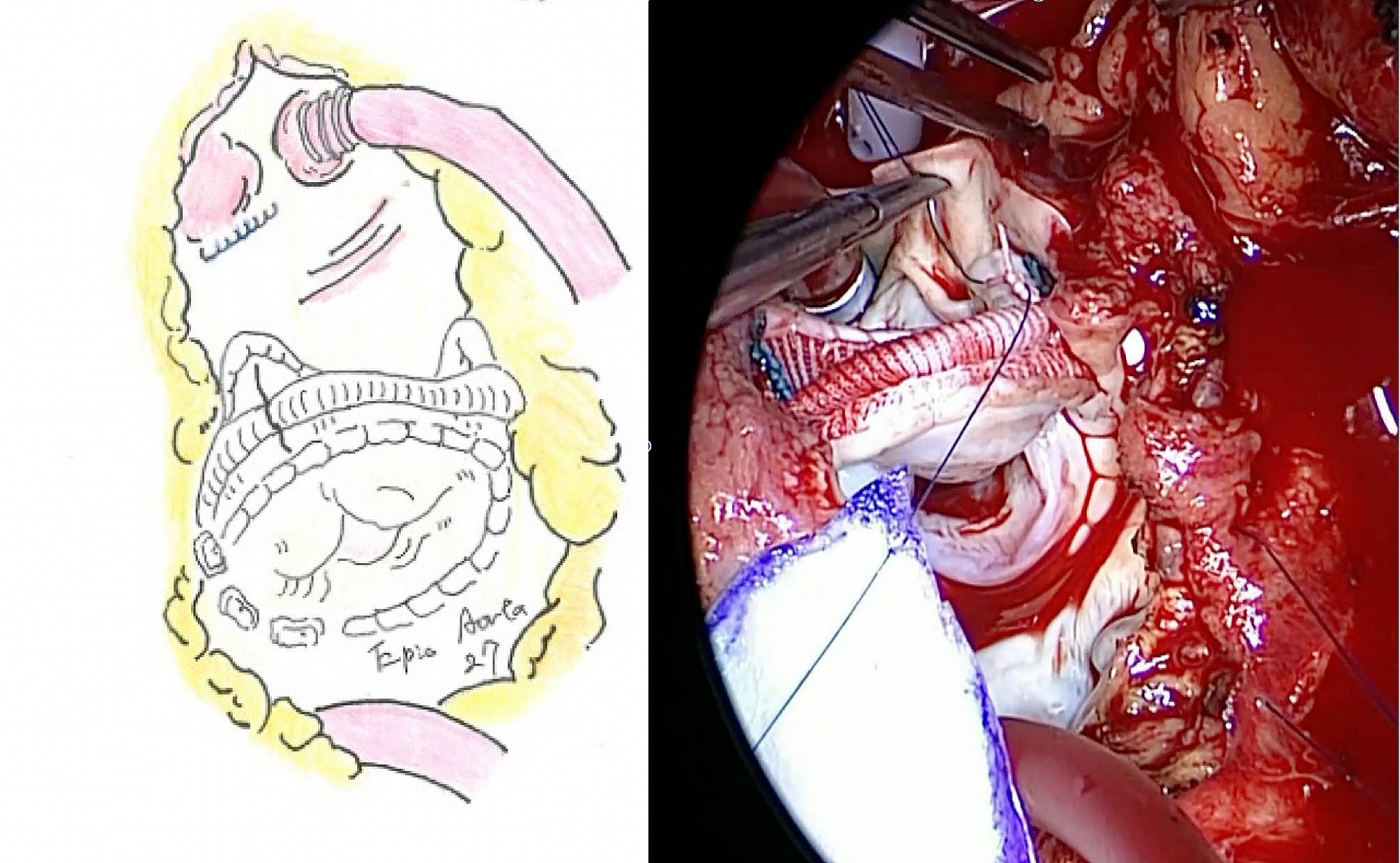
Pulmonary valve replacement (PVR) with annular enlargement using a bovine pericardium

**Fig. 2:**
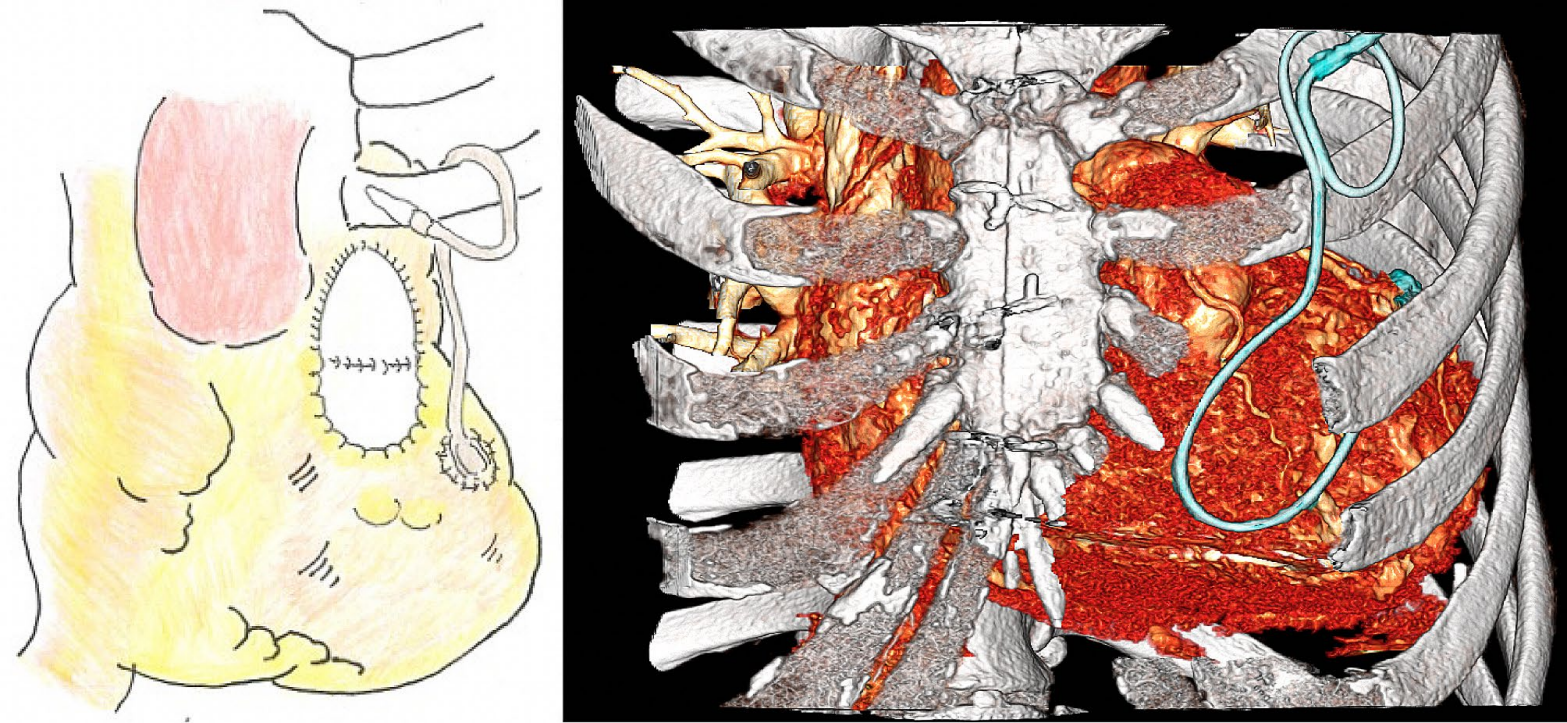
Epicardial lead placed at the left ventricular (LV) lateral wall mid to basal portion with the other end placed beneath the generator pocket of previous intravenous device through 3rd intercostal space

Second, we performed PVR with Epic™ Plus Supra Aortic Valve® (27 mm; Abbott Japan, Tokyo, Japan) and annular enlargement using a bovine pericardium (Fig. 1). In addition, pericardial lead, a sutureless, unipolar, myocardial, screw-in lead ( Medtronic Model 5071) was placed at the LV lateral wall mid to basal portion around the obtuse marginal branch region, with the other end placed beneath the generator pocket of the previous intravenous device through 3rd intercostal space (Fig. 2). No record was available in this case’s childhood surgery.　The operation took 6 h 56 min, with 233 min of cardiopulmonary bypass and 123 min of cross-clamp time.

Finally, 3 weeks after the surgery, CRT was adjusted using an epicardial lead in addition to intravenous defibrillator septal lead implantation. Postoperatively, echocardiography showed LVEF improved to 38% and LV shrank to LVDd/Ds 47 mm/31 mm. Furthermore, the symptoms of dyspnea on exertion resolved. Serum BNP was 89.1 pg/ml at the first visit, but had slightly decreased to 71.2 pg/ml one year after surgery. The ECG one after surgery showed a slight prolongation of QRS duration from 186 ms to 190 ms compared to preoperatively. Unexpectedly, one year after surgery, QRS fragmentation disappeared. These suggest that myocardial damage may have improved. QT prolongation was unchanged.

## Discussion and conclusions

In this case, PICM developed 5 years after TOF repair, and since a considerable period of time had passed since the repair, the patient had severe biventricularl heart failure. PiCM appears to occur in 10–20% of individuals within 3–4 years post PPM insertion. One recent study demonstrated that depending on the definition used, the incidence of PICM in a single cohort varied from 5.9% up to 39% [3]. In cases of severe biventricular failure, one of the representative current treatment strategies is heart transplantation. However, heart transplantation is not always the appropriate treatment for patients with adult congenital heart disease, due to relatively high mortality rates compared with those with normal anatomy [4, 5]. In the first place, we believe that heart transplantation is not necessarily the best initial treatment because it has side effects such as rejection and immunosuppressive therapy and requires a long waiting period. Alexander et al. reported that age > 42 years, atrial fibrillation, moderate QRS fragmentation, LVEF < 50%, RV end-diastolic pressure (RVEDP) > 16 mmHg, and LVEDP > 16 mmHg were independent risk factors for death and/or transplantation in adults with TOF [6]. In the present case, almost all of these risk factors, other than atrial fibrillation, were present. Because PVR increases the LV preload after surgery, preoperative medication with a combination of diuretics and sGC stimulator succeeded in reducing LVEDP.

In this case, the LV motion was almost paradoxical septal motion, that is, the septal wall deviated outward in systole synchronizes with lateral wall contraction; thus, a combination of left bundle branch pacing was expected in CRT. However, there are no long-term outcome data on CRT response in adults with repaired TOF in childhood [4]. The intravenous lead via the tricuspid valve carries the risk of TR progression and may be disadvantageous in the future. We considered removing the intravenous lead, but the preoperative CT suggested strong adhesion and the removal was not considered to be easy, so we decided to reuse it in this case.

To the best of our knowledge, the combination of epicardial LV lead and intravenous septal left bundle branch pacing in addition to PVR for biventricular failure after TOF repair has not been reported in the literature. In a patient with biventricular failure after TOF repair, a staged treatment strategy involving medication, PVR, and CRT with a combination of epicardial and intravenous leads could be a useful treatment worth trying before heart transplantation. However, if LV dysfunction progressed, it is important to not lose timing as a candidate for transplantation. Perioperative patient management including surgical strategy warrants further study.

## Data Availability

The datasets used and/or analyzed during the current study are available from the corresponding author on reasonable request.

## Abbreviations

TOF: tetralogy of Fallot
PR: pulmonary regurgitation
PVR: pulmonary valve replacement
LV: left ventricle
PiCMP: pacemaker-induced cardiomyopathy
CAVB: complete atrioventricular block
CRT: cardiac resynchronization therapy
LVEF: left ventricular ejection fraction
RV: right ventricle
RVFAC: right ventricular fractional area change
LVDd/Ds: left ventricular end-diastolic/end-systolic diameters
LVEDP: left ventricular end-diastolic pressure
sGC: soluble guanylate cyclase
RVEDP: right ventricular end-diastolic pressure
